# A framework for reconstructing transmission networks in infectious diseases

**DOI:** 10.1007/s41109-022-00525-4

**Published:** 2022-12-19

**Authors:** Sara Najem, Stefano Monni, Rola Hatoum, Hawraa Sweidan, Ghaleb Faour, Chadi Abdallah, Nada Ghosn, Hamad Hassan, Jihad Touma

**Affiliations:** 1 Department of Physics, American University of Beirut, Beirut, Lebanon; 2 Center for Advanced Mathematical Sciences, American University of Beirut, Beirut, Lebanon; 3 Department of Mathematics, American University of Beirut, Beirut, Lebanon; 4 Epidemiological Surveillance Program, Ministry of Public Health, Beirut, Lebanon; 5 National Center for Remote Sensing, National Council for Scientific Research (CNRS), Beirut, Lebanon; 6 Faculty of Public Health, Lebanese University, Beirut, Lebanon

**Keywords:** Network reconstruction, Betweenness centrality, Autoregressive model, COVID-19, Optimal control

## Abstract

In this paper, we propose a general framework for the reconstruction of the underlying cross-regional transmission network contributing to the spread of an infectious disease. We employ an autoregressive model that allows to decompose the mean number of infections into three components that describe: intra-locality infections, inter-locality infections, and infections from other sources such as travelers arriving to a country from abroad. This model is commonly used in the identification of spatiotemporal patterns in seasonal infectious diseases and thus in forecasting infection counts. However, our contribution lies in identifying the inter-locality term as a time-evolving network, and rather than using the model for forecasting, we focus on the network properties without any assumption on seasonality or recurrence of the disease. The topology of the network is then studied to get insight into the disease dynamics. Building on this, and particularly on the centrality of the nodes of the identified network, a strategy for intervention and disease control is devised.

## Introduction

Modeling the evolution of infectious diseases with the goal of forecasting the numbers of infections answers a wealth of questions in a range of disciplines from medicine, genetics, pharmacology to social and economic sciences [1, 2], and is at the heart of subsequent investigations on practical aspects: from management of resources (assessment of the preparedness for disease containment and readiness of the healthcare system) to possible intervention measures (vaccination and testing strategies [6], government control measures) and their consequences [7]. Some of these studies have a regional focus, investigating a disease propogation in countries, or regions of a country, while others have considered its dynamics in larger geographical contexts [3–5].

The different types of modeling that have been applied to investigate the dynamics of infectious disease have a long history in epidemic modeling. Compartmental models (e.g., SIR, SIER) are a well known class of models. They study the interplay between susceptible, infected and recovered individuals within communities, with different degrees of spatial refinement. For instance, in the so called networked compartmental models, interactions between communities are encoded in a network [8], often to identify the spatial and temporal origin of the disease [9]. In statistics, spatial/and/or temporal point processes are often employed to study the dynamics of the disease. Some models allow for the number of infections to be triggered by those at previous times, others can incorporate, as covariates, additional available information such as demographics, human mobility, and policy decisions. Some recent work follows this direction [10–12]. Epidemic models can also be recast in a standard regression framework, where the time series of infection counts are fitted by specifying a distribution for the counts and the associated conditional mean function [13, 14]. In most of these studies, the goal is to predict and capture the spatio-temporal patterns of disease spread. The major contribution of [13, 14] is in the study of diseases which exhibit a certain periodicity or recurrence, like influenza for example. Recently, this approach was implemented on COVID-19 data [3–5] to mainly break down the contributing factors to the disease spread. We observe that this model’s richness is not fully explored as it has an underlying time-evolving transmission network, which was never fully identified and whose properties have never been explored.

In this paper, we propose a generic framework for the recovery of the transmission network in infectious diseases. Our method does not assume any periodicity in the dynamics or any underlying recurrence. It hinges on the seminal work of [13, 14], which is a statistical regression analysis of infection counts over time and aggregated over localities, in a country, with a mean function that takes account of the spatial proximity of these localities. The fitted model is then used to reconstruct a weighted network, which constitutes the second component of our framework. The salient point of our method of network recovery is that smoothness conditions on the temporal data are not required [15], and neither is the near steady-state dynamics that is instead necessary for the perturbation/response approaches to work [16–18]. Our reconstruction is similar to that in [19], while it differs from procedures that rely on the deterministic evolution of the disease [20, 21]. The third component of our framework is the study of the changes in the topology of the underlying recovered network and the computation of centrality measures (specifically the nodes’ betweenness centrality), from which the recommendation of optimal control measures ensues. We apply the method in the case of COVID-19 in Lebanon.

## Model definition

Our starting point of the analysis is a statistical model that captures the spatio-temporal dynamics of the infections, under the statistical framework discussed in [13]. Namely, we consider the number $Y_{it}$ of infections recorded in a given locality *i* in a given day *t*, as independent, conditionally on the counts at previous times, random variables distributed according to a negative binomial distribution having a mean function decomposed into three terms as follows $\mu _{it} = E(Y_{i,t}|Y_{j,t-1}, e_i)$:

1 $$\begin{aligned} E(Y_{i,t}|Y_{j,t-1}, e_i) = \lambda _{it}Y_{i,t-1} + \phi _{it} \sum _{j \ne i} \omega _{ij}Y_{j,t-1} +\nu _{it}e_i. \end{aligned}$$

The first two terms constitute the auto-regressive part of the model: one being the contribution to the mean infection $\mu _{it}$ in locality *i* at time *t*, due to the infections within *i* at the previous day, the other being the contribution to $\mu _{it}$ due to positive cases from other localities *j* also at the previous day. The final term accounts for all other contributions not captured by the first two, such as infected people who entered the country under study from abroad. For simplicity we will refer to the last term $\nu _{it}e_i$ as the component due to travel and assume that it is proportional to the size of the population $e_i$ of the locality. The log-transforms of non-negative coefficients $\lambda _{it}$ and $\phi _{it}$, which quantify the contribution of the past observations to future counts, and the log-transform of the parameter $\nu _{it}$ are each modeled as a linear function of time, with a locality-specific slope to allow more flexibility across localities. Intercepts and slopes are estimated from the data. Finally, we model $\omega _{ji}$ as a power function of the geographical distance $d_{ij}$ of the localities: $\omega _{ji} \propto d_{ij}^{-f}$. This is assumed because previous studies have shown that mobility flows are governed by power-law functions of inter-localities distances [22–25].

## Network identification and characterization

We wish to focus now on the inter-locality term and study it from a different perspective. To do so, observe that the second term in the mean equation (1) can be re-written as follows: $\sum _{j \ne i}A_{ij}(t) Y_{j, t-1}$, where $A_{ij}(t)=\phi _{it}\cdot \omega _{ij}$. It can be interpreted as the contribution to the cases at time *t* in locality *i* from cases from locality *j* at the previous day. We can suggestively think of $A=(A_{ij})$ as defining the weights of a network between localities: the transport network $\omega$ describes the traffic flow between localities, and thus predates the disease, while $\phi _{it}$ is the number of transported cases from *i* into neighboring localities. *A*(*t*) explicitly depends on *t* since the coefficients of $\log \phi _{it}$, which are linear functions of *t*, and the power *f* in the definition of $\omega$ are estimated over each interval [0, *t*].

This complex network *A* drives the cross-localities dynamics. We will now suggest employing some useful summary metrics for *A*(*t*) and its time evolution in order to understand its properties, and accordingly prescribe adequate control measures.

**Fig. 1:**
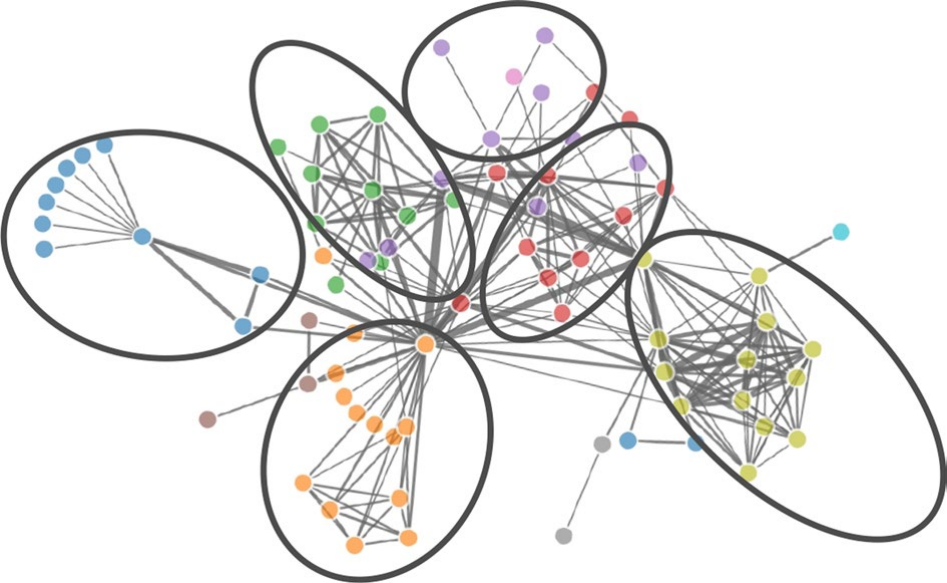
An example of modular network along with its detected communities (encircled) is shown. The nodes are color-coded based on their memberships to these communities

One useful summary metric is the modularity, which is a measure of cluster formation in a network. More specifically, the modularity *Q* of a given network *A* is defined with respect to a given grouping of its nodes. We follow [27] where the grouping of the nodes is determined by a stochastic procedure that reveals densely connected subgraphs. An illustration of a grouping is given in Fig. 1.

Given this group membership, the modularity of *A* is then computed according to the formula:

$$\begin{aligned} Q(A)=\frac{1}{2h} \sum _{{i,j}} \delta (c_i,c_j)(A_{ij}-k_i k_j/(2h) ) , \end{aligned}$$

where *h* denotes the total number of edges, $k_i$ and $k_j$ are the degree of nodes *i* and *j* respectively, $c_i$ labels the group to which *i* belongs, and $\delta$ is the Kronecker delta.

**Fig. 2:**
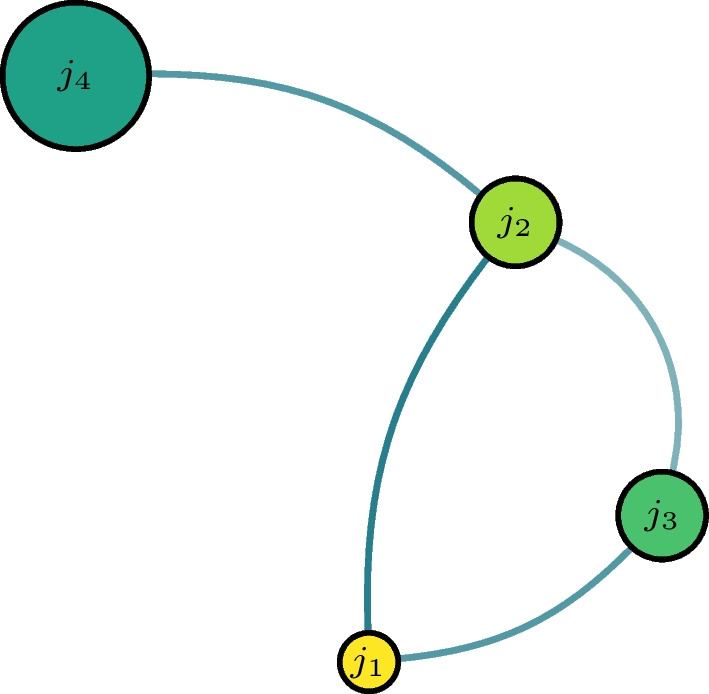
Example of a network with $C=1/3$ and $l = 8/6$ is shown. It has three triplets, one of which is closed (triangle). The lengths of the paths from $j_2$ to all others is 1, while that from $j_1$ to $j_4$ is 2

We also suggest to analyse additional topological measures for the networks: mainly, the clustering coefficient, the average path length , and the strength distribution [29, 30]. These are generally used to classify networks into random, scale-free, or regular. The clustering coefficient *C* of a network is a measure of transitivity that counts the ratio of the number closed triplets to the number of all (closed and open) triplets. A triplet is closed if all the three connections between the three nodes exist and is open if one of the links is missing. The average path length *l* of a network is given by the mean distance over all pairs of vertices, where distance is the number of edges in the shortest path joining them. An illustration is shown in Fig. 2.

Finally, a node’s strength is the sum of the weights of its edges. Namely, for the *i*-th node:

$$\begin{aligned} s_i = \sum _j A_{ij} \end{aligned}$$

**Fig. 3:**
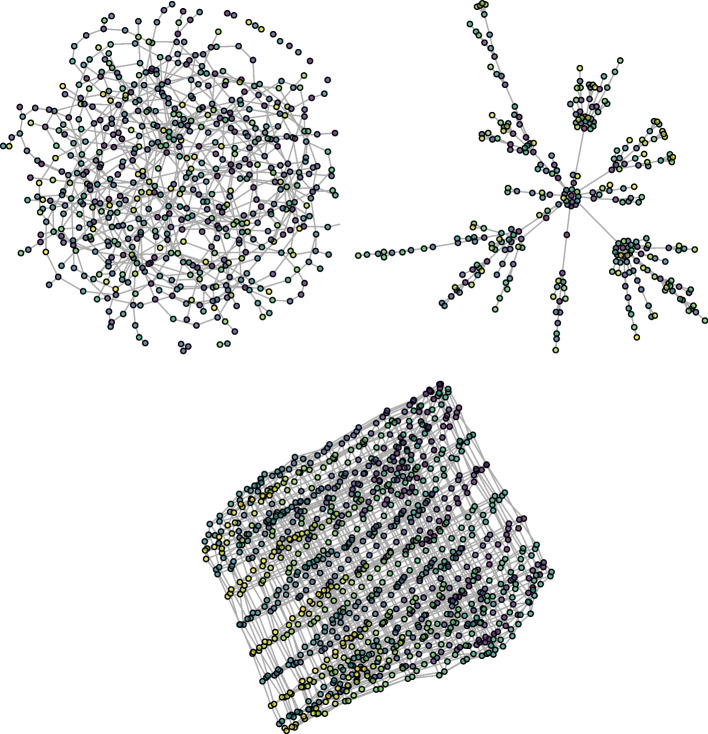
Examples of random, scale-free, and ordered (lattice-like) networks are shown respectively

Small-world or scale-free networks (that is, networks with node degrees and strengths distributed according to a power-law) are characterized by high clustering coefficients and low average path lengths compared with those of regular/ordered graphs [29, 30]. Random graphs are, on the other hand, characterized by low average path lengths and low clustering coefficients compared to regular graphs. An illustration of the three different network types is shown in Fig. 3.

## Data description and fitting of the regression model

As an application of the framework, the model (1) was applied to the COVID-19 data collected in Lebanon. On daily basis, the laboratories from the public and private sectors report the confirmed cases to the Epidemiological Surveillance Program of Lebanon’s Ministry of Public Health (ESUMOH). Later, the cases are investigated in order to get additional demographic information and health condition. The data are then archived in a national platform. Specifically, the data we have considered consist of counts of COVID-19 recorded daily in each of the 1544 localities of Lebanon from February 21, 2020 to January 20, 2022. Such localities correspond to Lebanon’s smallest statistical units called “circonscriptions foncières” or cadastral villages following the Central Administration for Statistics (CAS) nomenclature [26]. Recommendations on possible interventions and updates on the disease evolution were sought for by the Ministry of Public Health at 20 days intervals. Model (1) was fitted using the R package surveillance [14] over the intervals [0, *t*], $t=20 \cdot n$ for $n=1, \ldots , 41$. This allows us to follow the evolution in time of the model parameters until day 820, the last observation point.

**Fig. 4:**
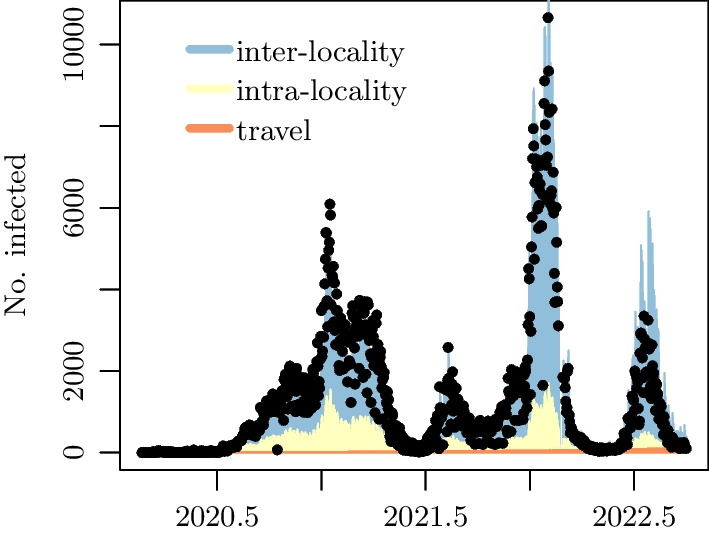
Data and fit under the model of Eq. (1) with negative binomial distributed counts. The three colors show the decomposition of the fitted aggregate counts into travel, intra-locality, and inter-locality contributions to infections amounting to $3\%$, $10\%$, and $87\%$ respectively

Figure 4 displays the aggregated counts over all localities, $\sum _{i} Y_{i, t}$, and the fitted values over the complete time period of our study broken down into the three components of the mean function. The fit appears quite adequate. It is in fact a better fit to the data than the model with counts assumed to be Poisson-distributed, which is an indication of overdispersion in the data. A further comparison of these two models in terms of AIC value and prediction errors is provided in Table 1.

**Table 1:** Comparison of the two models with the same mean function given in Eq. (1) with distribution of the counts being negative binomial and Poisson

	ses	AIC
Negative binomial	2.88	1,164,119
Poisson	3.65	1,333,453

AIC is Akaike’s Information criterion. SES is the mean squared error: the mean squared difference over the localities of the observed and predicted counts at the final time point of the study

We further notice that the model of equation (1) considers a time-lag of one day; that is, the future counts depend on the counts recorded on the previous day. Changes in the time-lag from one to a few days did not result in any noticeable difference. Our analysis provides evidence that the inter-locality infection drives the overall transmission of the disease [22]. Then, for this reason we shift our focus to the network that governs the interaction between localities and observe that it is not purely a static spatially-dependent network but rather dynamic and time-evolving: in fact the product of time-dependent coefficients with the spatial proximity matrix. Figure 4 indicates that the inter-locality term has the most important contribution to the increase in the mean number of infections compared to the intra-locality and travel terms. This suggests that the inter-locality transmissions should be the main focus of analysis, and what one learns from their study would be useful for disease control.

**Fig. 5:**
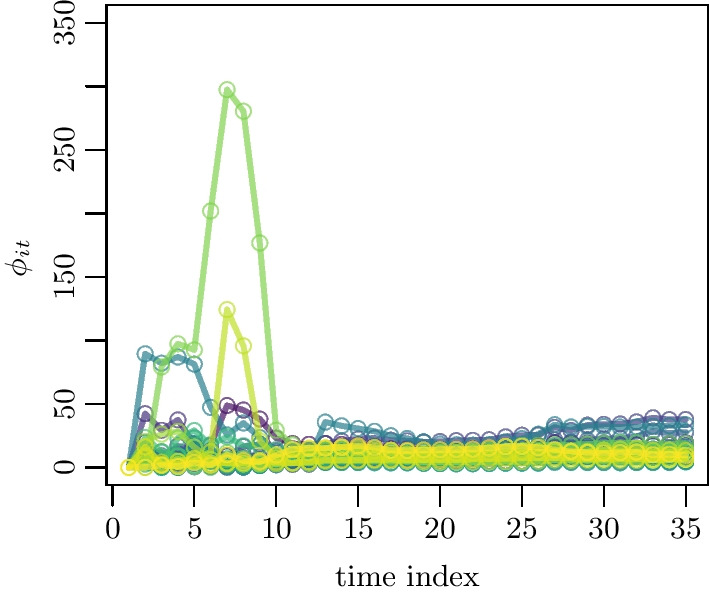
Time evolution of the inter-locality parameter $\phi _{it}$ is shown for the regions with the highest centrality (see Sect. 3)

The parameter estimates of model (1) for all 1554 localities and their errors can not be displayed in an uncluttered fashion but are available from the corresponding authors. A sample of the evolution of the inter-locality term $\phi _{it}$ is shown in Fig. 5.

**Fig. 6:**
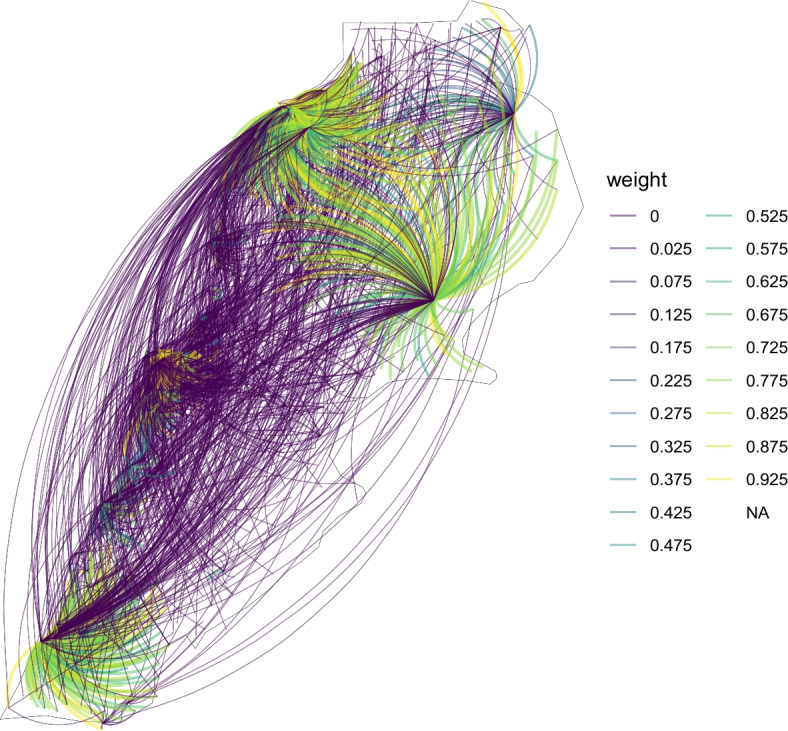
The network $A^{(15)}(t=300)$ is overlaid on the map of Lebanon to illustrate its complexity. It is evaluated at the 300-th day of the pandemic using all 15 time interval of our collected data, that is the time span $[0,20\cdot 15]$

An example of the reconstructed network is provided in Fig. 6 which is a graphical representation of $A^{(15)}(t=300)$. The superscript 15 in the notation of *A* indicates that the latter was estimated on the counts data of 15 contiguous 20-day time intervals, that is the 300-day time span $[0,20\cdot 15]$ from February 21, 2020 to December 16th, 2020.

**Fig. 7:**
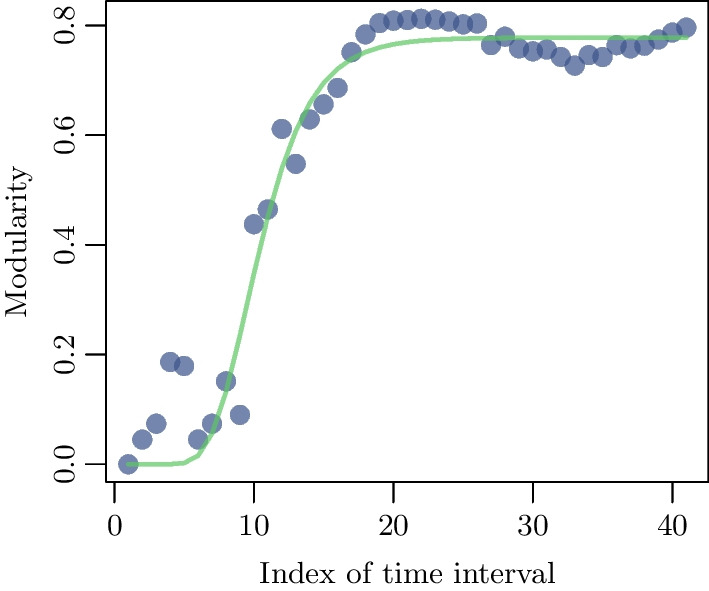
The modularity of the network $A^{(n)(t)}$ is shown as a function of time $t = 20 \cdot n$, with $n=1 \ldots 41$. It is measure of the quality of the division of a graph into subgraphs

Further, Fig. 7 shows the modularities of the 41 matrices $A^{(41)}(t)$ at days $t=20 \cdot n$ for $n=1, \ldots , 41$. The superscript *n* in the notation $A^{(n)}(t)$, as mentioned above, indicates that *A* is estimated using the counts of the $20\cdot n$ days of the study.

**Fig. 8:**
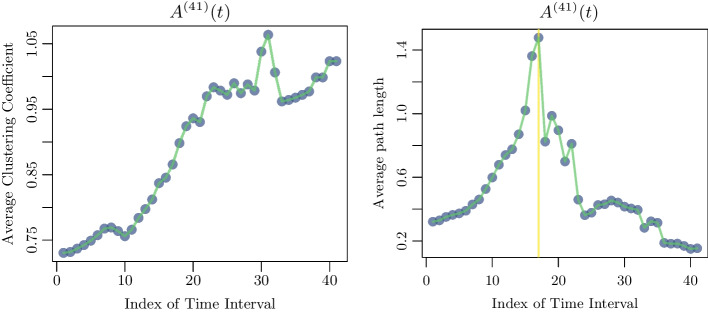
The figure shows the clustering coefficient and average path length for $A^{(41)}(t)$, which are the matrices estimated using the data from the start of the pandemic to the 820-th day ($41 \cdot 20$) evaluated at $t = 20 \cdot n$, where *n* is the index of the time intervals

Figure 8 shows the clustering coefficients and the average paths lengths for the matrices $A^{(41)}(t)$. Similar behavior of both *C* and *l* was observed for all $A^{(n)}$, with $n \ge 10$.

One can see a jump in modularity on the tenth 20-day time interval, which we will denote by $I_c$. This behavior may signal the onset of an emerging power-law [28]. The evolution of both *C* and *l* gives additional evidence for a transition at a point $I_c$. The clustering coefficient starts suddenly to increase. At the 10-th interval there is an abrupt jump in the average path length as well at $I_c$ (Fig. 8). This is an indication of scale-freeness of the network. This property expedites the spread of epidemics unlike what would occur in ordered networks, which are characterized by a slower spread because they possess a high *C* and an *l* that scales with system size [31, 32].

**Fig. 9:**
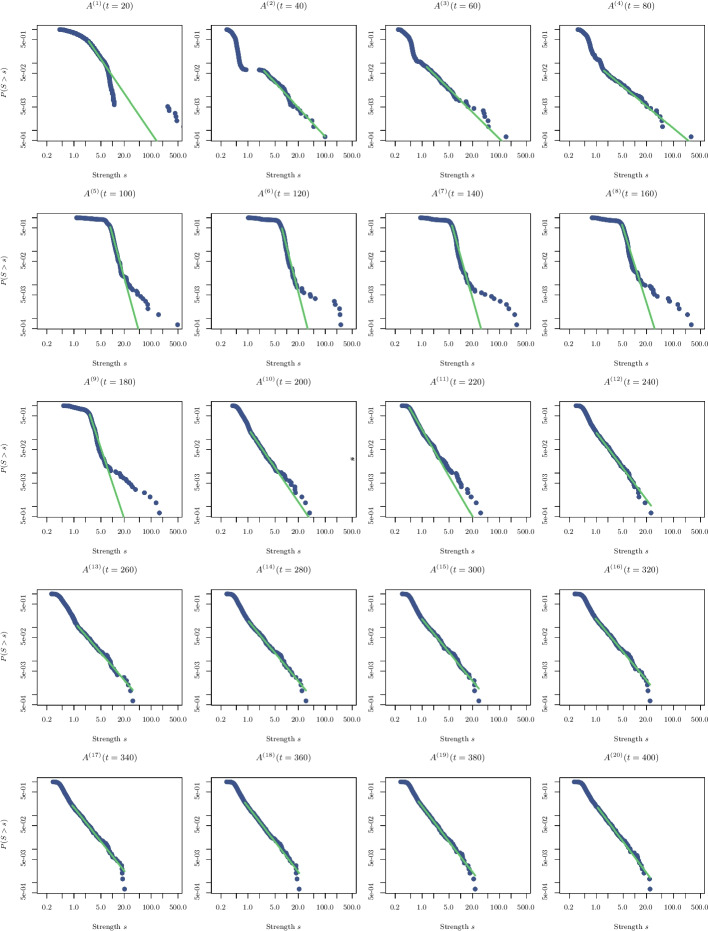
Empirical and estimated distributions of the strength for the matrices $A^{(n)}(t = 20\cdot n)$ with $n=1, \ldots , 20$ are shown

**Fig. 10:**
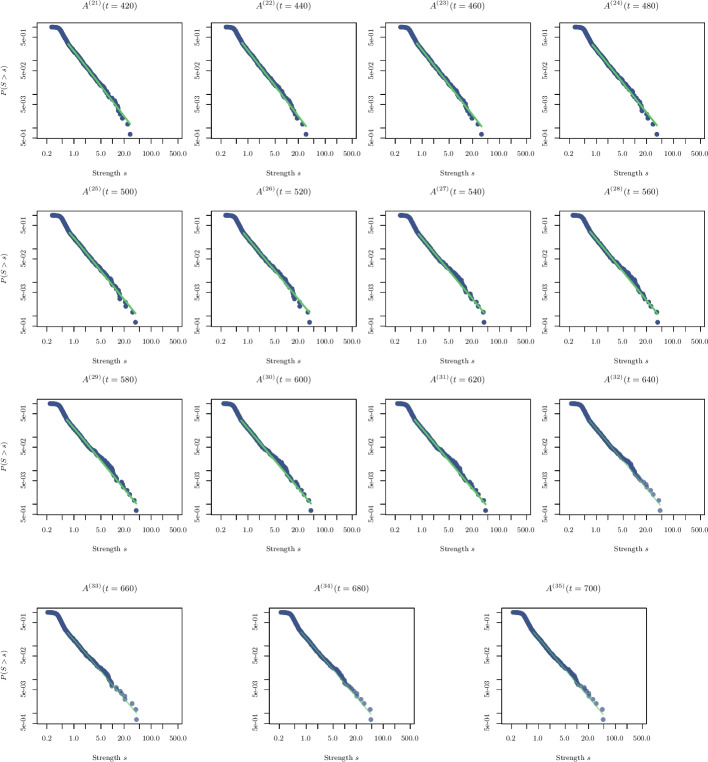
Empirical and estimated distributions of the strength for the matrices $A^{(n)}(t = 20\cdot n)$ with $n=21, \ldots , 35$ are shown

To characterize the transition to scale-freeness, we now analyse the distribution of the strengths of the nodes, as additional evidence for change in the network topology at the 10-th interval $I_c$. Figures 9 and 10 show the empirical and estimated distributions of the strengths (in fact, the survival function $P(S> s)$) of $A^{(n)}(t = 20\cdot n)$, at the time intervals $n=1, \ldots , 41$ on a log-log scale. We note that a transition occurs at $t=20 \cdot 10$, where the distribution becomes linear, which is indicative of a power-law (Pareto distribution): $P(X \le x)=1-(\beta /x)^{\alpha -1}$, for $x\ge \beta$. The exponent $\alpha$ and the boundary value (scale) $\beta$ are estimated by maximum likelihood following [33].

**Fig. 11:**
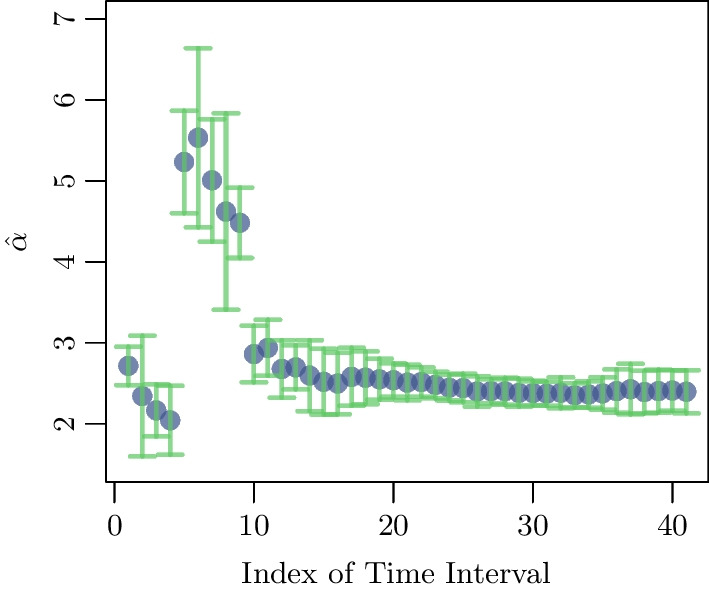
Estimated values ${\hat{\alpha }}$ of the exponent of the power-law distributions of the strengths of the nodes of the 41 matrices $A^{(n)}(t = 20\cdot n)$, $n=1, \ldots , 41$, which are represented in Fig. 9. Vertical bars indicate $\pm 2 {\hat{\sigma }}_{{\hat{\alpha }}}$

Figure 11 summarizes the estimates of the exponents for these networks and their standard errors (obtained by non-parametric bootstrap).

After the 180th day, that is for time intervals labeled by the index $n \ge 10$, most power-laws have very close exponents of about 2.5. This signals the stabilization of the network topology. Thus, $I_c$ marks the onset of the emergence of the steady state network. We think that only above this point any prescription of control measures is likely to be efficient as the revealed network topology, relying on the daily counts, has stabilized. One can wonder if there is any explanation on why the stable phase has set in during this interval $I_c$, and not before or after it. $I_c$ chronologically coincides with the period between August, 19, 2020, and September 7, 2020. Perhaps, the blast in Beirut which occurred on August 4th and in the following weeks of social protests, personal precaution measures (such as social distancing and wearing of masks) were compromised. Either of these occurrences may have contributed to the detected change in the network type. See the Appendix for the chronology.

## Putting the analysis into action: control measures

**Fig. 12:**
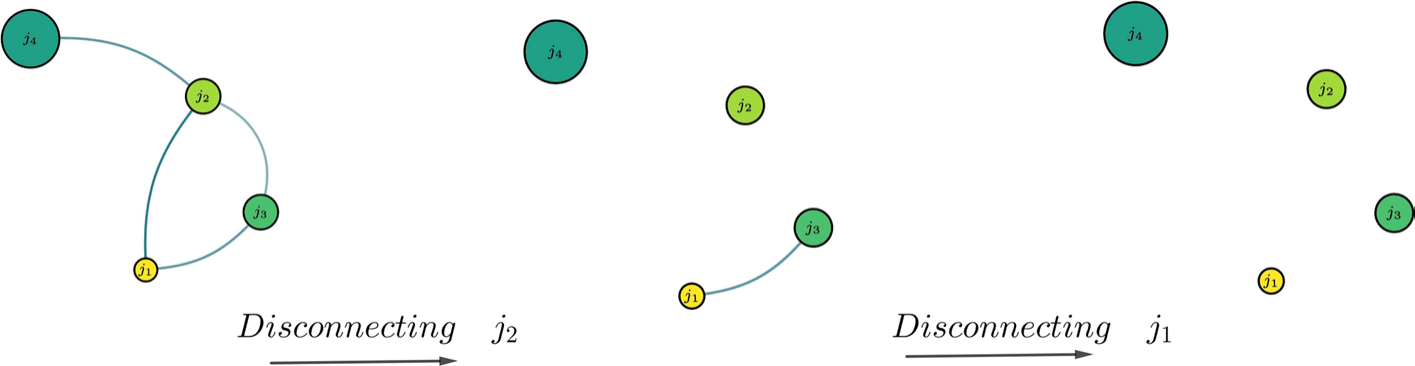
The figure illustrates the iterative scheme. First, the node with the highest centrality $j_2$ is disconnected by removing all its links, as it is the node with the highest number of shortest paths. The resulting network has $j_1$ and $j_3$ with equal centralities and either one can be disconnected. This leads to a total loss of connectivity in the network at the end of the process

Having fully characterized the network and identified the steady-state, we now turn to a possible use of this analysis to guide an optimal strategy for disease control. The strategy will identify some localities as candidates for being isolated or for having their connections to other localities curtailed. The measure on which the identification is based is that of centrality of a node. The betweenness centrality of a node *v* is defined as [30]:

$$\begin{aligned} g(v)=\sum _{{i\ne v\ne j}}{\frac{\sigma _{{ij}}(v)}{\sigma _{{ij}}}} \end{aligned}$$

where $\sigma _{ij}$ is the total number of shortest paths from node ${\displaystyle i}$ to node ${\displaystyle j}$ passing through $\sigma _{ij}(v)$. Therefore, the more central the node is, the more its removal has an effect on the network’s connectivity, since its removal would yield a network with more disconnected subgraphs. The control strategy we propose involves an iterative procedure, where at each step the centralities of the nodes are computed, the node with the resulting highest centrality is removed, and the matrix *A* is updated, as illustrated in Fig. 12.

**Fig. 13:**
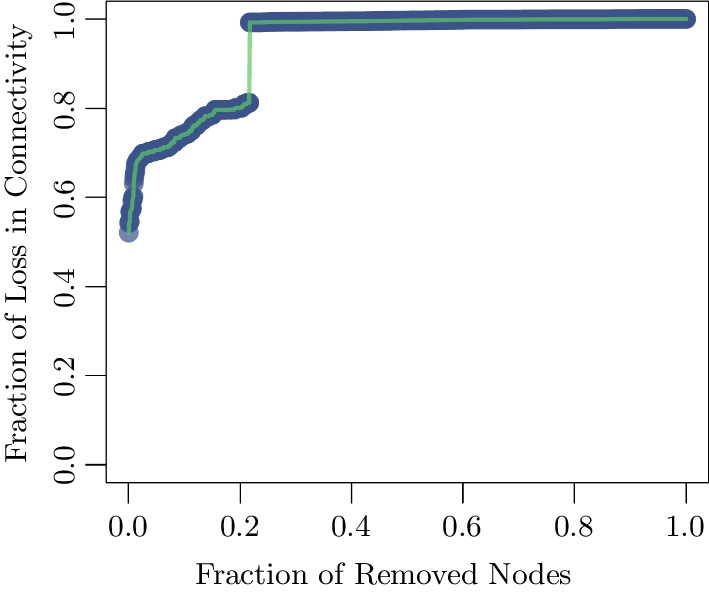
The loss of connectivity versus the fraction of removed nodes for the cascading and non-cascading strategies

Other removal schemes of nodes in network exist, but the one we have just described has been suggested to incur the highest loss of connectivity for scale-free networks [34–39]. In practice, candidate targets for intervention the localities corresponding to nodes with higher centralities. We notice that at the policy level this strategy based on our analysis was indeed adopted. The localities we have identified through this strategy were given priority in the national vaccination campaign. On the other hand, the recommendations we put forward based on this analysis were only partially adopted in targeting the high centrality localities for lockdown and intervention measures, as the decision making process involved other ministries and stakeholders. However, we conclude by considering theoretically the would-be repercussions of such implementation. Clearly, the loss of connectivity would impede the evolution of the disease since the localities which are contributing the most to the infection would be isolated. For example, removing around $20\%$ of the most connected localities on the basis of their betweenness centrality would lead to $80\%$ loss of connectivity as shown in Fig. 13.

**Fig. 14:**
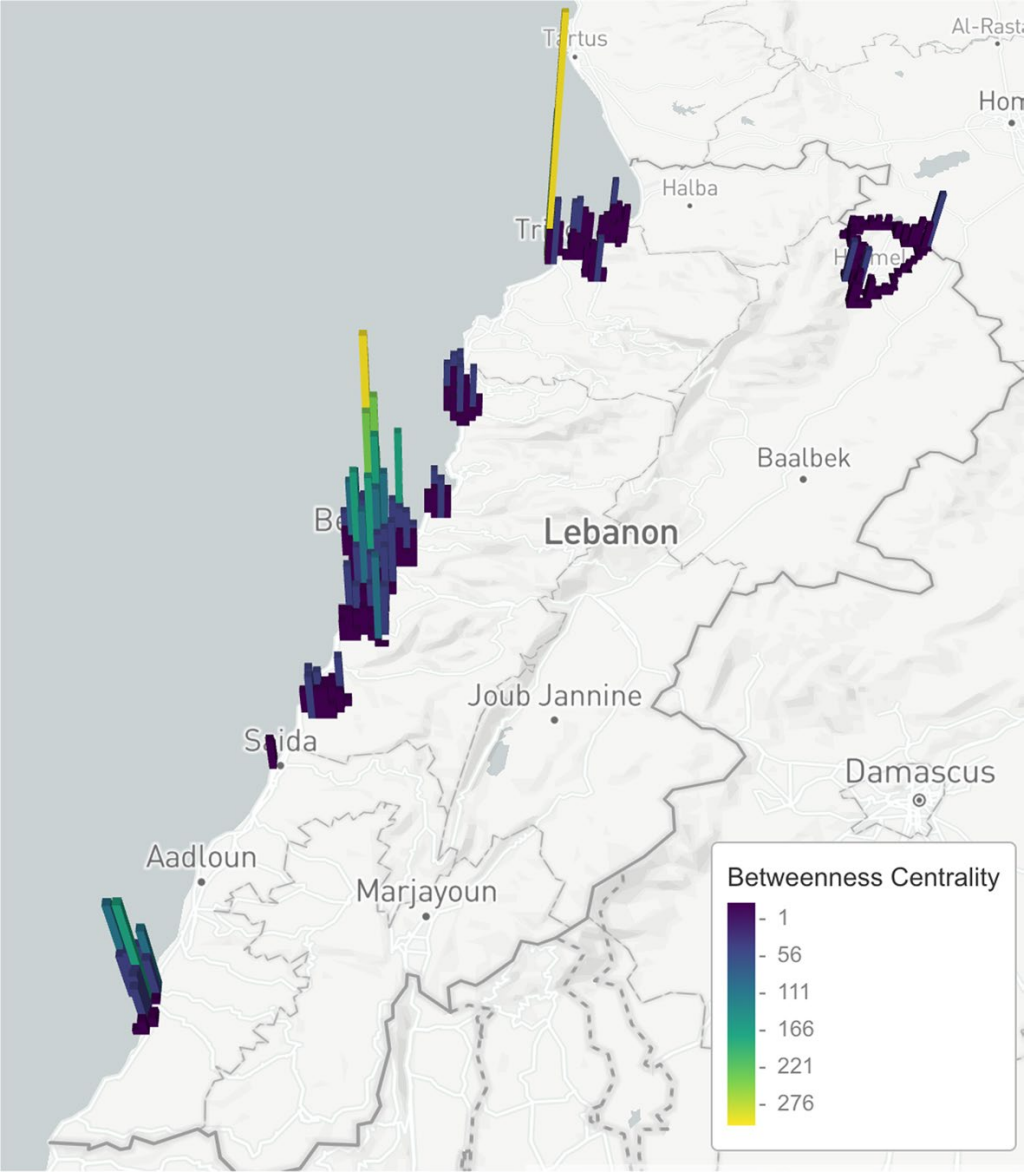
The map shows the localities with the highest centrality whose removals lead to $80\%$ loss of connectivity

**Fig. 15:**
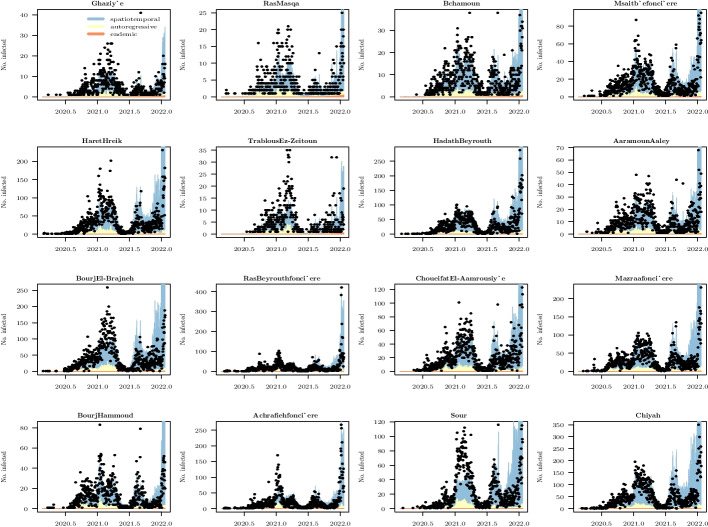
The figure shows the counts, along with the fitted model, for twelve localities ordered by decreasing betweenness centrality

Specifically, the localities causing $80\%$ loss of connectivity are shown in Fig. 14, while the fitted model of the top sixteen localities is shown in Fig. 15. An animated map of the control strategy is available on this https://www.dropbox.com/s/guhamz3p7op6b3y/animated.gif?dl=0.

## Conclusion

In this paper, we have proposed a framework that can be used to inform control measures for epidemics in a country for which infections counts aggregated over local regions are available over time. In particular, and as an example, we have followed the evolution of the counts of COVID-19 cases in Lebanon at the level of local administrative units at a daily resolution. The framework entails fitting an auto-regressive model to the data; recovering an underlying network over which the disease propagates; analyzing such time-evolving network to identify topological measures of node centrality that suggest an optimal control of the spread of the disease. Specifically, for the data about COVID-19 in Lebanon the analysis of the topological metrics of the network has given us a hint into a transition to a steady state structure that governs interactions between localities. After identifying this steady state network, and characterizing it as a scale-free, we have proposed control measures based on betweenness centrality of its nodes. The findings were taken into consideration in the national vaccination campaign for COVID-19, with the identified localities given priority for vaccination.

## Data Availability

The data is available upon request from the Ministry of Public Health’s Surveillance Program.

## Appendix: Chronology of COVID-19 pandemic in Lebanon

In this appendix we summarize the chronology of the COVID-19 pandemic in Lebanon from the first recorded case to February 2022. We divide this time interval into 4 periods, and highlight the main governmental interventions taken to control the spread of the disease.

Period 1 (February 2020 to June 2020). The first cases are documented. Early lockdown measures are implemented with airport closure. Testing is carried out for suspected cases, close contacts, and travelers. Cases are mainly within clusters. Aggressive contact tracing is adopted.

Period 2 (July 2020 to December 2020): The airport reopens in July 2020. The daily number of cases increases progressively and community transmission sets in. On August 4th, the Beirut blast occurs.

Period 3 (January 2021 to June 2021): The alpha variant is introduced. The case counts increase. Lockdown measures are implemented resulting in a decrease of the recorded cases. However, after lockdown release, an increase of the number of infections is observed until mid-March, with a progressive and sustained decrease up to June.

Period 4 (July 2021 to December 2021). Introduction of the delta variant, which progressively replaces the alpha variant. Two waves of delta are observed: July–September and November–December.

Period 5 (January 2022 to February 2022). Introduction of the omicron variant. High transmissibility of the new variant leads to high daily case counts reaching 10,000 on 1st Feb 2022.

## Abbreviations

ESUMOH: Epidemiological Surveillance Program of Lebanon’s Ministry of Public Health
CAS: Central Administration for Statistics
